# Epigenetics and Preeclampsia: Defining Functional Epimutations in the Preeclamptic Placenta Related to the TGF-β Pathway

**DOI:** 10.1371/journal.pone.0141294

**Published:** 2015-10-28

**Authors:** Elizabeth Martin, Paul D. Ray, Lisa Smeester, Matthew R. Grace, Kim Boggess, Rebecca C. Fry

**Affiliations:** 1 Department of Environmental Sciences and Engineering, Gillings School of Global Public Health, 135 Dauer Drive, CB 7431, University of North Carolina, Chapel Hill, North Carolina, United States of America; 2 Curriculum in Toxicology, School of Medicine, University of North Carolina, Chapel Hill, North Carolina, United States of America; 3 Department of Obstetrics & Gynecology, University of North Carolina School of Medicine, University of North Carolina, Chapel Hill, North Carolina, United States of America; CNRS, FRANCE

## Abstract

Preeclampsia is a potentially fatal pregnancy disorder affecting millions of women around the globe. Dysregulation in gene and protein expression within key biological pathways controlling angiogenesis has been implicated in the development of preeclampsia. Altered CpG methylation, a type of epimutation, may underlie this pathway dysregulation. In the present study, placental tissue from preeclamptic cases and normotensive controls was analyzed for genome-wide differential CpG methylation and concomitant changes in gene expression. A set of 123 genes, representing 19.9% of all genes with altered CpG methylation, was associated with functional changes in transcript levels. Underscoring the complex relationships between CpG methylation and gene expression, here hypermethylation was never associated with gene silencing, nor was hypomethylation always associated with gene activation. Moreover, the genomic region of the CpG mark was important in predicting the relationship between CpG methylation and gene expression. The 123 genes were enriched for their involvement in the transforming growth factor beta (TGF-β) signaling pathway, a known regulator of placental trophoblast invasion and migration. This is the first study to identify CpG hypomethylation as an activator of TGF-β-associated gene expression in the preeclamptic placenta. The results suggest functional epimutations are associated with preeclampsia disease status and the identified genes may represent novel biomarkers of disease.

## Introduction

Across the globe preeclampsia is associated with the deaths of ~76,000 women and ~500,000 fetuses each year and impacts 5–8% of pregnancies in the United States alone [1]. Characterized by maternal hypertension and proteinuria that can progress to organ failure, seizures, and maternal death, preeclampsia presently has no viable treatment or prevention options [2]. A precise etiology of preeclampsia is unknown but is linked to impaired vascular development/angiogenesis of the placenta, or “poor placentation,” hypothesized to be the primary pathological mechanism underlying the disease phenotype [3]. The causal factors for poor placentation are presently unknown and likely multi-factorial in nature [2].

A possible etiologic factor in preeclampsia is an imbalance between angiogenic and antiangiogenic growth factors. Angiogenic growth factors such as vascular endothelial growth factor (VEGF) and placental growth factor (PLGF) are required for invasion of the spiral artery and proper placentation [2]. Inhibitors of VEGF, such as soluble Fms-related tyrosine kinase 1 (sFLT-1), have been implicated in the etiology of preeclampsia [2]. Additionally, sFLT1 is the best studied of these growth factors and has been proposed for use as a potential clinical biomarker of preeclampsia as it is highly expressed in the serum of women with preeclampsia [4], particularly at early gestational ages [5]. By binding VEGF and PLGF, sFLT-1 is thought to create an anti-angiogenic environment preventing proper invasion of the maternal spiral arteries [6]. Still, it is unclear why women who develop preeclampsia have higher levels of sFLT-1, and at the present time it is not used in the clinical setting [7]. These data highlight a gap in knowledge of the contribution of specific genes that underlie preeclampsia.

Epimutations, or epigenetic modifications such as DNA methylation, are known drivers of molecular changes to gene and protein expression levels [8]. Additionally, epimutations (specifically, alterations CpG methylation) can be induced by environmental factors [9]. Genome-wide hypomethylation in preeclampsia has been previously observed [10,11]. As CpG methylation can directly influence the expression of genes and subsequently proteins, it has the potential to be a major contributor to disease.

The relationship between specific genes with altered CpG methylation in the preeclamptic placenta and altered functional changes in transcript level is understudied. Prior studies have examined differentially methylated genes in preeclampsia, and have compared changes in genes with differential CpG methylation in genes to publicly available gene expression data [11–15] but only one has simultaneously assessed both genome-wide DNA methylation levels and genome-wide mRNA transcripts in the same samples [10]. To begin to fill this knowledge gap, we analyzed relationships between changes in CpG methylation in placental tissue from women with preeclampsia compared to controls, and the associated functional changes in gene expression levels. Transcription factor binding site analysis was used to identify potential key regulators of the CpG methylation patterning in the preeclamptic placenta.

## Methods

### Study design and collection of placental tissue samples

A total of 36 women (17 normotensive controls and 19 preeclamptic cases) receiving obstetric care at UNC hospitals consented to collection of placental samples at the time of birth. Subjects gave written informed consent to being enrolled and participating in this study. Preeclamptic subjects were identified as displaying sustained *de novo* hypertension (>140/90 mmHg) and proteinuria (>300mg of protein in a 24 h urine collection or urine protein/creatinine ratio of 0.3mg/dL), developing after 20 weeks of pregnancy. For inclusion in the study, subjects had to have either blood pressure >160/110 mmHg, severe neurologic complications, or lab abnormalities consistent with HELLP (hemolysis, elevated liver enzymes, low platelet count) in addition to being identified as having preeclampsia syndrome as defined by the American Congress of Obstetricians and Gynecologists. Women with confounding conditions such as pre-diabetes, diabetes, and gestational diabetes were excluded from the study. A full-thickness placental biopsy was obtained after delivery, avoiding the periphery and areas of obvious infarction, flash frozen in liquid nitrogen, and subsequently stored at -70°C until analysis. This research was approved by the Institutional Review Board at the University of North Carolina (#11–2054).

### CpG methylation and gene expression analysis

A 0.2 g subsection of placental tissue was cut from each frozen biopsy on dry ice, washed briefly in sterile 1X PBS to remove any residual blood, and homogenized in Buffer RLT with B-mercaptoethanol (Qiagen, Valencia CA). DNA and RNA sequences greater than 18 nucleotides in length were collected using the AllPrep DNA/RNA/miRNA Universal Kit (Qiagen, Valencia CA) according to manufacturer’s instructions. CpG methylation was assessed using the Illumina HumanMethylation450 BeadChip array (Illumina, Inc., San Diego, CA). This platform assesses the DNA methylation levels of 486,428 individual probes at single nucleotide resolution. Isolated DNA was first bisulfite-converted using the EZ DNA methylation kit (Zymo Research, Irvine, CA) and converted DNA was then hybridized onto the array. The DNA methylation data were collected at Expression Analysis, Inc. (Durham, NC; www.expressionanalysis.com). Methylation levels were calculated and expressed as β values (β = intensity of the methylated allele (M) / (intensity of the unmethylated allele (U) + intensity of the methylated allele (M) + 100). RNA abundance was analyzed using the Affymetrix GeneChip ® Human Gene 2.0 ST array as described previously [16]. All data are available at the Gene Expression Omnibus (GEO) (GSE73377).

### Statistical analysis

To analyze CpG methylation levels, the data were pre-processed, following the pipeline described in Morris et al., 2014 before performing statistical analysis [17]. For the primary analysis to identify preeclampsia-associated CpG methylation, probes that represent known single nucleotide polymorphism (SNPs) were removed (n = 89,678) as the technology cannot be used to distinguish between sequence-based differences and CpG methylation differences [18]. However, to allow for cross-study comparability we also conducted a secondary analysis to determine whether any of the SNP-associated probes showed significant differences in CpG methylation between preeclamptic placentas and controls. To control for the quality of probes in the analysis, probes with detection p-values > 0.01 (n = 6,298) were removed from analysis. Data were quantile normalized to remove experimental artifacts inherent to the array. The resulting 390,452 quantile normalized probes were assessed for differential methylation in relation to preeclampsia case status using an Analysis of Covariance (ANCOVA) model controlling for gestational age, maternal age, and race [7]. The ANCOVA analysis was conducted with Partek Genomic Suite 6.4 (St Louis, Missouri). Parametric and nonparametric analyses were conducted using SAS 9.3 (SAS Institute Inc., Cary, North Carolina) to determine the relationship between demographic variables and preeclampsia status and for covariate selection. Differential DNA methylation levels were statistically defined as: a p-value <0.01 for associations with case status with a false discovery rate (FDR) corrected q-value < 0.1. To determine if CpG methylation was associated with gene expression, Pearson correlation coefficients and associated p-values were calculated with significance set at p<0.05. We also performed a complementary analysis to enable cross-study comparisons, of all genes with differential expression between the preeclamptic and normotensive placentas. The differentially expressed genes were identified using an Analysis of Covariance (ANCOVA) model controlling for gestational age, maternal age, and race as detailed above. Differential gene expression was defined as: a p-value <0.01 for associations with case status, false discovery rate (FDR) corrected q-value < 0.1, and fold change ≥ 2 or ≤-2.

While single nucleotide polymorphisms were removed (n = 89,678) from the primary analysis, to allow for cross-study comparability we conducted a separate analysis to determine whether any of the SNP-associated probes were likewise differentially methylated in relationship to preeclampsia. Thus, all SNP-associated probes were examined for their relationship to preeclampsia with a total of n = 389 identified to be differentially methylated between the preeclamptic and normotensive placentas (S1 Table).

### Gene ontology and pathway enrichment analysis

Genes with differentially methylated CpG levels associated with case status were analyzed for enrichment of canonical pathways using Ingenuity Pathway Analysis Software (Ingenuity Systems, Inc., Redwood City, CA). Gene networks representing homeostatic and pathological processes were identified through enrichment analyses. Statistical significance of network enrichment was determined as previously described [19]. P-values represent the probability of differentially methylated genes appearing in the associated biological network at random. String bioinformatics software was used to construct protein networks from genes determined to be associated with the enriched canonical pathways [20].

Additionally, we set out to determine whether there was an enrichment of transcription factor binding sites among the genes with differential CpG methylation. Using the Gene2Promoter module Genomatix (http://www.genomatix.de) [21], the promoter region sequences of each of the genes within the TGF-β pathway were analyzed with the Common Transcription Factor Analysis Module. Core similarities of 95%, and the presence of the consensus sequence in more than 50% of the analyzed genes were required. P values, representing the likelihood of obtaining an equal or greater number of sequences with a match in a randomly drawn sample of the same size as the input sequence set, are reported.

## Results

### Description of the Cohort

A total of 36 women were represented in the study, including 17 normotensive and 19 preeclamptics. Most of the women were of African American decent representing 35.3% of normotensive women and 47.4% of preeclamptic women. Most of the women were primipara (58.3%) and most were non-smokers (92.5%) (Table 1). The median age was 28.4 among women with preeclampsia and 28.2 among normotensive women. Of the demographic variables tested, only gestational age differed between the two groups. Women with preeclampsia delivered significantly earlier than normotensive women (32.8 vs 38.6 weeks, p = 0.0001) (Table 1).

**Table 1:** Demographic characteristics of the study subjects.

Characteristics	All N (%) /Mean (Range)	Controls(Normotensives) N (%) /Mean (Range)	Cases(Preeclamptics) N (%) /Mean (Range)	p-value
**Subjects**	36 (100.0%)	17 (47.2%)	19 (52.8%)	
**Race**				0.196
** African American**	15 (41.7%)	6 (35.3%)	9 (47.3%)	
** Asian**	1 (2.8%)	1 (5.9%)	0 (0.0%)	
** Caucasian**	11 (30.6%)	6 (35.3%)	5 (26.3%)	
** Hispanic**	6 (16.7%)	4 (23.5%)	2 (10.5%)	
** Other**	3 (8.3%)	0 (0.0%)	3 (15.8%)	
**Parity**				0.519
** Primipara**	21 (58.3%)	9 (52.9%)	12 (63.2%)	
** Multipara**	15 (41.7%)	8 (47.1%)	7 (36.8%)	
**Smoking Status**				0.548
** Smoker**	2 (7.5%)	1 (5.9%)	1 (5.3%)	
** Non-Smoker**	34 (92.5%)	16 (94.1%)	18 (94.7%)	
**Maternal Age (years)**	/28.3 (19–38)	/28.2 (19–38)	/28.4 (19–37)	0.9809
**Gestational Age (weeks)**	/35.5 (22–41)	/38.6 (35–41)	/32.8 (22–40)	0.0001*

* p value < .05

### Differential CpG methylation and functional changes in gene expression levels in the preeclamptic placenta

The methylation status of 390,452 distinct CpG probes corresponding to 20,452 individual genes was analyzed across all placentas. There were 989 differentially methylated probes (DMP) between the preeclamptic and normotensive placentas. As more than one probe can correspond to a single gene, these probes represented 617 differentially methylated genes (DMGs) (Table 2, S2 Table). The majority (n = 689, 69.7%) of the DMPs displayed a difference in CpG methylation that exceeded 5% (Δβ ≤ -0.05 or Δβ ≥ 0.05) with a maximum difference in methylation of ~20% (Δβ = -0.198, Δβ = 0.191) (S2 Table). In terms of directionality, most (80.7%) of the DMPs were hypomethylated in the preeclamptic placenta versus the normotensive placenta while only 19.3% were hypermethylated (Table 2, S2 Table). The five most significant DMGs that were hypermethylated in the preeclamptic placentas relative to normotensive placentas were Protein Tyrosine Phosphatase, ReceptorType, N Polypypeptide 2 (*PTPRN2*, Δβ = 0.191), Olfactory Receptor, Family 1 Subfamily I, Member 1 (*OR1I1*, Δβ = 0.174), Myelin Oligodendrocyte Glycoprotein (*MOG*, Δβ = 0.165), Melanoma Antigen Family B6 (*MAGEB6*, Δβ = 0.151), and Relaxin Family Peptide Receptor (*RXFP*, Δβ = 0.149). The five most significant DMGs that were hypomethylated in the preeclamptic placentas relative to normotensive placentas included Methylenetetrahydrofolate Dehydrogenase (NADP+ Dependent) 1-Like (*MTHFD1L*, Δβ = -0.194), Beta- Transducin Repeat Containing (*BTRC*, Δβ = -0.186), Dystonin *DST* (Δβ = -0.185), Melanocortin 1 Receptor (*MC1R*, Δβ = -0.174) and Zinc Finger, Swim-type (*ZSWIM4*, Δβ = -0.173).

**Table 2:** Methylation status for differentially methylated probes (DMPs) or functionally- changed differentially methylated probes (fDMPs).

Methylation Status	DMPs (n = 989)	fDMPs (n = 132)
**Total Probes**	989	132
**Hypermethylated**	191 (19.3%)	18 (13.6%)
** Positively Correlated (Activation)**	-	18 (100.0%)
** Negatively Correlated (Silencing)**	-	0 (0.0%)
**Hypomethylated**	798 (80.7%)	114 (86.4%)
** Positively Correlated (Silencing)**	-	5 (4.4%)
** Negatively Correlated (Activation)**	-	109 (95.6%)

**Fig 1:**
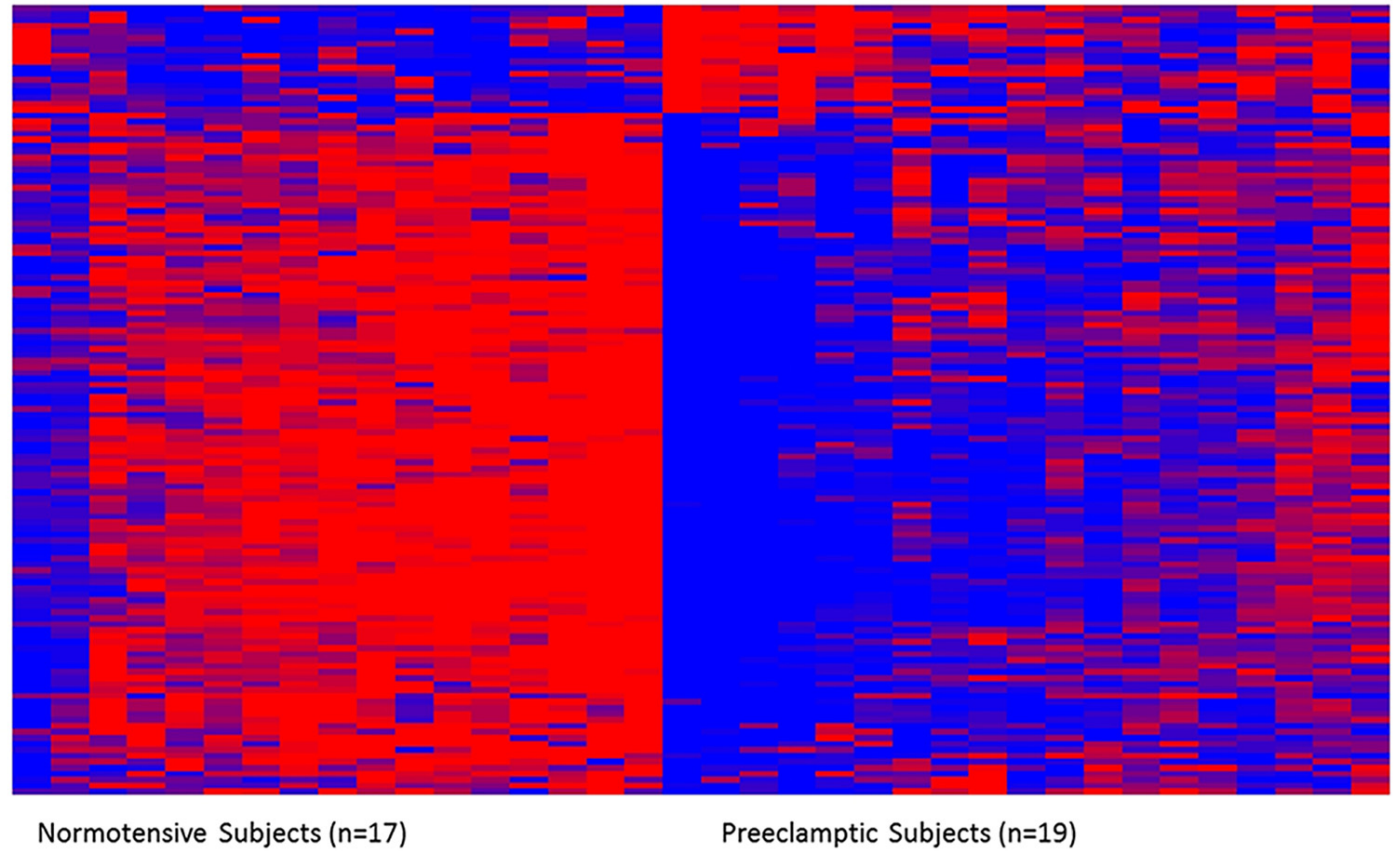
The heatmap displays the relative methylation levels of the 132 DMPs across subjects. Preeclampsia is associated with differential methylation and expression (not shown) of 132 differentially methylated probes (DMPs) representing 123 unique genes (fDMGs). Methylation levels are *z*-score normalized across rows; red indicates relatively higher levels of methylation and blue indicates relatively lower levels of methylation**.**

The 989 DMPs were matched to their corresponding genes and subsequently analyzed for association with gene expression across all subjects. A total of 695 DMPs could be matched between the CpG methylation and gene expression platforms. These analyses revealed 132 DMPs (n = 123 genes) with a significant (p<0.05) association between CpG methylation level and gene expression level representing functionally-changed differentially methylated probes (fDMPs) (S3 Table, Fig 1). This number of fDMPs/fDMGs represents a limited proportion of the total DMPs/DMGs, 13.3% of probes and 19.9% of genes respectively. Similar to the DMPs, the majority (86.4%) of fDMPs were hypomethylated in the placentas of preeclamptic women versus normotensives, with only 13.6% were hypermethylated (Table 2, S3 Table). Correlation coefficients for the relationships between CpG methylation and gene expression ranged from −0.77 to 0.67, with 32 of the 132 DMPs displaying a higher than moderate (R≥0.5) correlation (S3 Table).

**Fig 2:**
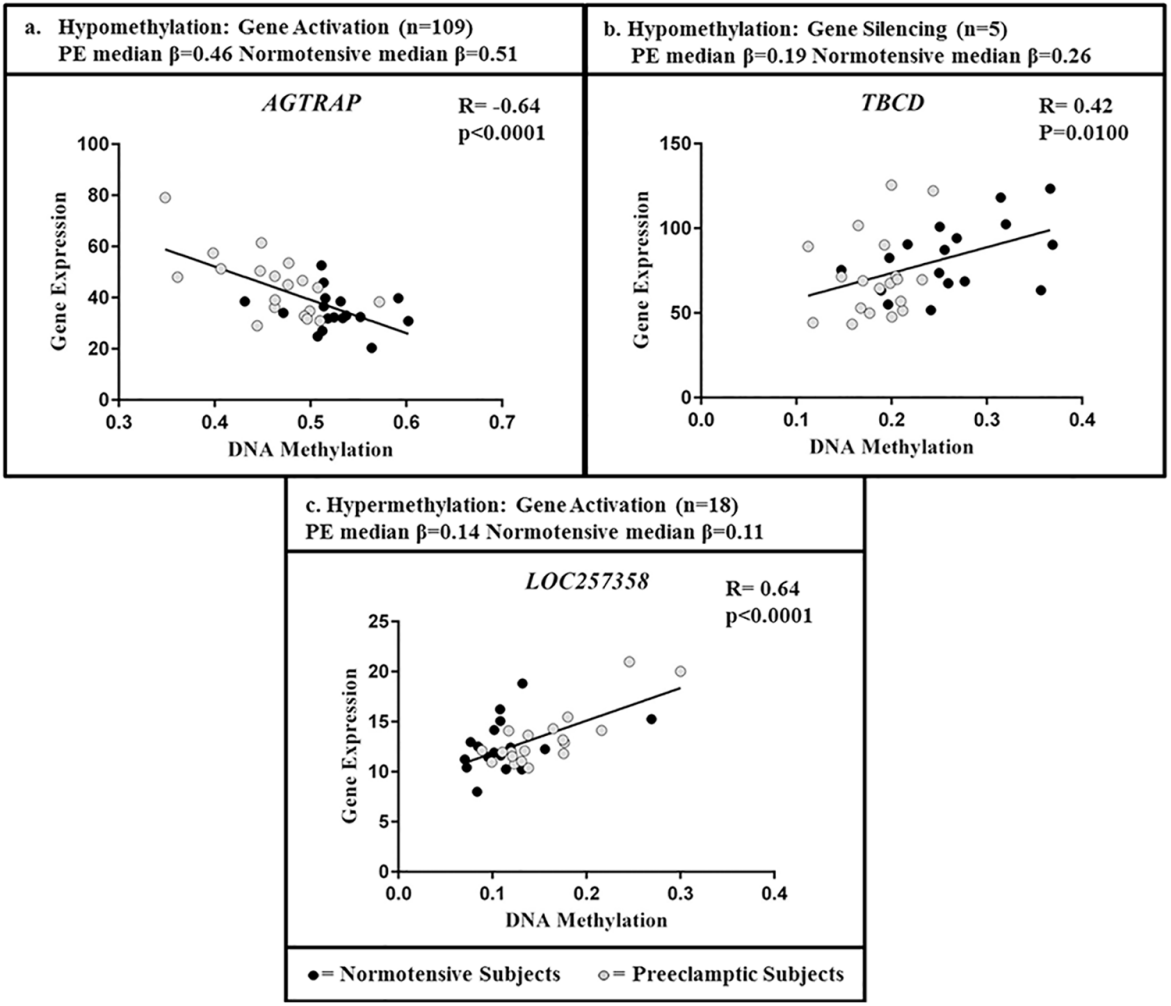
Plots of CpG methylation levels and gene expression levels across all subjects for three representative genes. The plots display the three observed relationships of CpG methylation and gene expression. For each plot, the median β value for the preeclamptic and normotensive subjects is provided. The total number of genes (n) identified in the study that display a similar pattern as the representative genes is detailed.

Hypermethylation is often described as a silencer of gene expression while hypomethylation is associated with gene activation [22]. In line with this, in the placentas studied here, the majority (95.6%) of hypomethylated probes were associated with gene activation displaying a negative correlation between CpG methylation and expression. In contrast to this, 4.4% of the hypomethylated probes were associated with gene silencing (Table 2, S3 Table). Genes displaying these relationships are shown in Fig 2, with angiotensin receptor associated protein, *AGTRAP*, representative of a gene with hypomethylation and gene activation, and tubulin folding cofactor D, *TBCD*, representative of a gene with hypomethylation and gene silencing (Fig 2A and 2B). Surprisingly, all of the hypermethylated fDMPs were associated with gene activation, with a positive correlation between CpG methylation and expression (Table 2, S3 Table). Representative gene *LOC257358*, displays this relationship of gene hypermethylation and increased gene expression (Fig 2C).

### Position-based analysis reveals a relationship between the location of CpG methylation and subsequent gene expression patterns

The Illumina HumanMethylation450 BeadChip array contains information detailing the genomic positions of the CpG probes within each gene. These are classified into six positions: 1500 base pairs upstream of the transcription start site (TSS1500), 200 base pairs upstream of the transcription start site (TSS200), gene body (Body), the 5’ untranslated region (5’ UTR), the 3’ untranslated region (3’ UTR), and the 1^st^ exon (1^st^Exon). As the position of a methyl mark within a gene has been previously been shown to be associated with gene expression [23], we set out to determine whether CpG position predicted functional changes in gene expression in the placenta.

DMPs and fDMPs were not clustered in specific genomic regions, rather they were evenly distributed across the six different regions tested (Table 3). Region-based analysis demonstrated that many (44.4%) of the hypermethylated marks occurred in gene bodies. Similarly, most (50%) of the hypomethylated marks also occurred within the gene bodies, with 20% occurring within the TSS1500 (Table 3).

**Table 3:** Number and percent of differentially methylated probes (DMPs) or functionally-changed differentially methylated probes (fDMPs) identified based on genomic region. Hypermethylation (hyper) and hypomethylation (hypo) in relationship to preeclampsia are detailed.

**Region**	**Probes Tested (n)**	**DMPs (% Probes Tested)**	**fDMPs (% DMPs)**	**Hyper fDMPs (% hyper-fDMPs)**	**Hypo fDMPs (% hypo-fDMPs)**
**1st Exon**	18,609	36 (0.19%)	6 (17%)	3 (17%)	3 (3%)
**3'UTR**	13,738	36 (0.26%)	7 (19%)	0 (0%)	7 (6%)
**5'UTR**	35,131	83 (0.24%)	19 (23%)	3 (17%)	16 (14%)
**Body**	129,162	338 (0.26%)	65 (19%)	8 (44%)	57 (50%)
**TSS1500**	56,943	148 (0.26%)	24 (16%)	1 (6%)	23 (20%)
**TSS200**	42,544	54 (0.13%)	11 (20%)	3 (17%)	8 (7%)
**Unspecified**	94,325	294 (0.31%)	0	0	0
**Total (n)**	390,452	989 (0.25%)	132 (13.3%)	18 (100%)	114 (100%)

### Relationship to previously implicated preeclampsia-associated genes

Through literature reviews we identified a total of 376 genes suspected to play a role in preeclampsia (S4 Table). Of these, 25 were among the 617 DMGs representing a significant (χ^2^ p-value = 0.0002) number and seven were fDMGs (S2 Table, S3 Table). Six of the seven displayed expression changes that were consistent with what has been previously reported in the literature: angiotensin receptor associated protein (*AGTRAP)*, collagen Type IV Alpha 2 (*Col4A2*), glyceraldehyde 3-phosphate dehydrogenase (*GAPDH*), krueppel-like factor 9 (*KLF9*), lactate dehydrogenase A (*LDHA*), and solute carrier family 1 (Neutral Amino Acid Transporter), member 5 (*SLC1A5*) transcripts (S3 Table). The expression of plasma membrane calcium-transporting ATPase 4 (*ATP2B4*) was associated with preeclampsia, however the relationships reported were not consistent between studies. In the present study it was found to be hypomethylated in preeclamptic women, as well as increased in expression (S3 Table).

### The TGF-β pathway is enriched among the functionally changed DMGs

**Fig 3:**
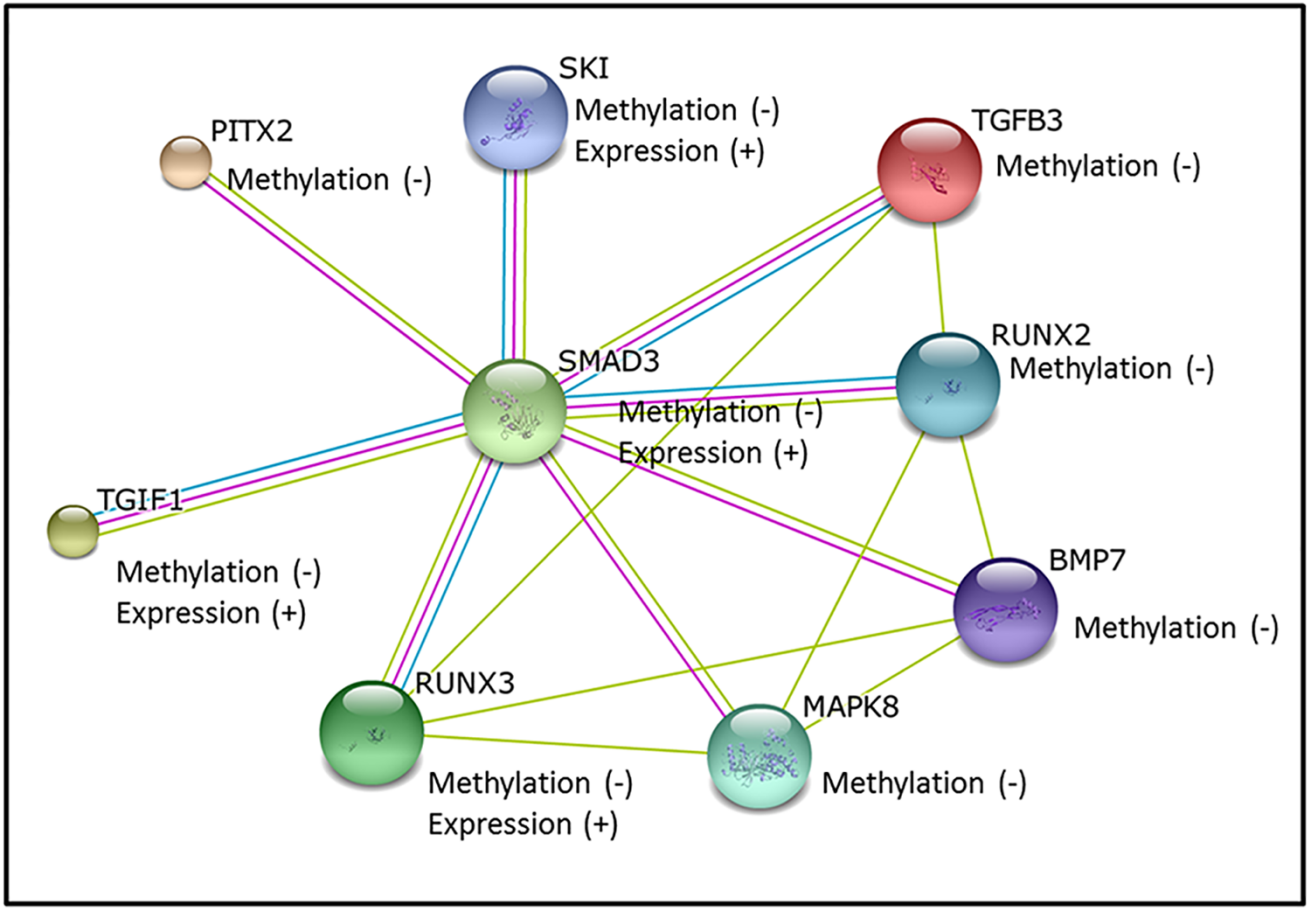
Protein interaction network showing the relationships between differentially methylated genes within the TGF-β pathway. The colors represent the different lines of evidence: green represents associations determined by literature searches, magenta represents experimentally-derived evidence, and blue represents associations reported in databases.

All DMPs and DMGs, both those that were only changed at the CpG methylation levels and those that were functionally changed at the gene expression level, were analyzed for their role in biological pathways. Interestingly, the TGF**-**β pathway was significantly enriched within both the DMGs (p = 0.0005) and fDMGs (p = 0.0014) (S5 Table). The following five genes were differentially methylated: Runt-Related Transcription Factor (*RUNX2*) (Δβ = -0.112), Mitogen Activated Protein Kinase 8 (*MAPK8*) (Δβ = -0.076), Transforming Growth Factor- β 3 (*TGFβ3*) (Δβ = -0.068), Bone Morphogenic Protein 7(*BMP7*) (Δβ = -0.105), and Paired-like homeodomain 2 (*PITX2*) (Δβ = -0.077). The following four genes were differentially methylated and differentially expressed (fDMGs): Runt-Related Transcription Factor 3(*RUNX3*) (Δβ = -0.109), Mothers against decapentaplegic homolog 3 (*SMAD3*) (Δβ = -0.162), proto-oncogene Ski (*SKI*) (Δβ = -0.120), and TGFB-induced factor homeobox 1 (*TGIF1*) (Δβ = -0.078). The TGF-β-associated genes were exclusively hypomethylated with some displaying concomitant increases in gene expression (Fig 3).

Analysis of enrichment for binding sites for transcription factors among the differentially methylated genes that were associated with the TGF-β pathway was conducted. This analysis revealed enrichment for binding sites for three transcription factors with enriched binding sites in the sequences: Kidney-enriched Kruppel-like factor 15 (KLF15), Myeloid Zinc Finger Protein 1 (MZF1), and Kruppel-like Zinc Finger protein 219 (ZNF219) (Table 4).

**Table 4:** Transcription factors with enriched binding sites among the TGF-β pathway-associated genes.

**Gene Symbol**	**Full Transcription Factor Name**	**p-value**	**Total # Sequences**	**# Matched Sequences**
***KLF15***	Kidney-enriched kruppel-like factor 15	0.001304	68	24
***MZF1***	Myeloid zinc finger protein 1	0.001968	41	25
***ZNF219***	Kruppel-like zinc finger protein 219	0.000288	73	24

Also of interest were the fDMPs associated with Tetrahydrofolate Salvage from 5,10-methenyltetrahydrofolate and Folate Metabolism (S5 Table). These pathways include two genes: Methylenetetrahydrofolate Dehydrogenase (NADP+ Dependent) 1-Like (*MTHFD1L*) and Methylenetetrahydrofolate Dehydrogenase (NADP+ dependent) 2 (*MTHFD2*), both of which were hypomethylated with increased expression (S2 Table). To note *MTHFD1L* was the most hypomethylated gene in the dataset, with 20% lower methylation in the preeclamptic placenta relative to the normotensive placenta (S2 Table, S3 Table).

### Identification of differentially expressed genes independent of changes in methylation status

As a supplemental analysis to enable cross-study comparisons, genes with altered gene expression in the preeclamptic placenta were identified irrespective of changes to CpG methylation. This results in the identification of 1,602 probes corresponding to 729 unique differentially expressed genes (DEGs) (S6 Table). Comparisons to genes implicated in preeclampsia revealed that 13 out of the 729 genes with altered expression were among the 381 genes that have been previously associated with preeclampsia, an insignificant number (p = 0.852) (S6 Table, S3 Table).

### Relationship to previously implicated gestational age-associated genes

While the statistical model used to identify probes with significant differences in CpG methylation associated with preeclampsia corrected for gestational age of the subjects, we wanted to ensure that the differences in CpG methylation were not simply due to differences in gestational age of the subjects. Gene expression data from Winn et al., were compiled representing 418 genes known to have differential expression in the placenta dependent upon gestational age [24]. The DMPs and fDMGs that were identified were compared to this list and only 15 preeclampsia-associated DMPs were identified (p = 0.112). The DMGs associated with gestational age were: Melanocortin 1 recptor (*MC1R*), Lactate Deyhdrogenase (*LDHA*), CoA Synthase (*COASY*), Ras Related Protein (*RAB31*), Adipocyte Adhesion Molecule (*ASAM*), Paired-like Homeodomain 2 (*PITX2*), RNA Binding Motif Single Stranded Interacting Protein 1 (*RBMS1*), PDZ and LIM Domain 1 (*PDLIM1*), Solute Carrier Family 35 (*SLC35D1*),Tumor Necrosis Factor Receptor Superfamily Member 21 (*TNFRS21*), Dipeptidase 2 (*DPEP2*), Ciracdian Associated Receptor of Transcription (*C1orf151*), Von Willebrand Factor (*VWF*), Ral Guanine Nucleotide Dissociation Stimulator-like 2 (*RGL2*), and V-ETs Avian Erythoroblastosis Virus E26 Oncogen Homolog (*ERG*). Only one of these genes, *PITX2*, was among the genes belonging to the TGF-β pathway. Only five of the fDMGs were significantly associated with gestational age (p = 0.160). These included *TNFRSF21*, *VWF*, *SLC35D1*, *LDHA*, and *PDLIM1* and none of these genes were members of the TGF-β pathway (S3 Table).

## Discussion

The molecular mechanisms underlying the initiation and disruption of placental gene expression observed in preeclampsia remain largely unknown. Here we identified sites of CpG methylation altered in the preeclamptic placenta, as well as CpG methylation marks that were directly linked to functional changes in gene expression in the preeclamptic placenta. The majority (69.7%) of the CpG methylation changes identified in the preeclamptic placenta displayed greater than a 5% difference relative to normotensive subjects, a change that has been associated with functional implications for altered gene expression [25,26]. Most of these observed differences in CpG methylation were lower in the preeclamptic placenta relative to the normotensive placenta supporting previously published data regarding hypomethylation of promoters regions in the preeclamptic placenta [10,11]. In addition to the identification of CpG methylation related to the TGF-β pathway, we identified 25 known preeclampsia-associated genes with altered CpG methylation with seven displaying functional changes in gene expression including *AGTRAP* and *Col4a2*. *AGTRAP* plays a role in the angiotensin pathway and *Col4a2* is associated with early onset preeclampsia [2,7]. These data highlight that there is a general loss of CpG methylation in the preeclamptic placenta that is significantly different from normotensive women, and that gene expression is tied to these changes in methylation.

Interestingly, genes with altered CpG methylation and concomitant changes in gene expression level enrich for the TGF-β pathway and include: *SMAD3*, *SKI*, *RUNX3*, and *TGIF1*. For all genes associated with the TGF- β pathway, the methylation differences between the preeclamptic and normotensive placentas exceeded 5%. The TGF-β pathway showed a general hypomethylation with increased expression in the preeclamptic placenta. Supporting our results, this pathway has been implicated in control of trophoblast invasion and migration. TGF-β is elevated in the serum of preeclamptic patients and is known to inhibit the angiogenic factor VEGF in many tissues [27–29]. In the present study *SMAD3* (Δβ = -0.162) was among the top 10 most hypomethylated sites and displayed increased expression in the preeclamptic placenta. *SMAD3* is known to complex with TGF- β [30,31]. While not identified to differential CpG methylation in the present study, an additional member of this pathway, TGF- β2, has been proposed as a predictive biomarker for preeclampsia with elevated serum levels in women with preeclampsia [32]. In the present study TGF- β3 was hypomethylated (Δβ = -0.068) in the preeclamptic placenta which is interesting as increased expression of TGF- β3 during pregnancy has been associated with preeclampsia [33]. This is the first study to highlight the epigenetic regulation of the TGF-β signaling as a potential etiologic factor in preeclampsia.

Our hypothesis for gene-specific patterning of DNA methylation is the “transcription factor occupancy” theory where binding of transcription factors can influence subsequent methylation patterning across the genome [23,34]. Interestingly, two of the three transcription factors associated with the TGF-β pathway-associated genes have been previously associated with preeclampsia. Both ZNF219 and MZF-1 have demonstrated altered expression in the decidua or placentas of preeclamptic women [35,36]. While KLF15 has not previously been identified in association with preeclampsia, it is a known regulator of TGF- β expression [37,38]. Taken together these data suggest that transcription factor binding may influence the DNA methylation patterning related to TGF- β in the preeclamptic placenta.

It was interesting to find fDMGs that play a role in folate scavenging metabolism, including *MTHFD1L* and *MTHFD2*, that showed hypomethylation and increased expression in the preeclamptic placenta. Additionally, among all genes tested, *MTHD1L* displayed the greatest degree of change in methylation, with a nearly 20% decrease in CpG methylation in the preeclamptic placenta relative to the normotensive placenta. These genes have not been previously implicated in preeclampsia or associated with preeclampsia, however, folate deficiency has been previously tied to preeclampsia [39]. It is thought that the lack of folate may be tied to the incorrect development of the placenta [39]. Supplementation with folate has been used with limited success in attempts to decrease risk for preeclampsia [40,41]. Interestingly both of the genes we identified with increased expression encode enzymes linked to the conversion, and thus depletion, of folate to tetrahydrofolate and its downstream metabolites.

CpG methylation and gene expression correlations are typically described as being negative where hypermethylation acts as a silencing mechanism and hypomethylation an activating mechanism [22]. In the present study, the majority of fDMPs were negatively associated with gene expression. There were a number of fDMPs, however, that displayed a positive association between CpG methylation and expression. We observed that hypermethylation within the gene body was often associated with increased gene expression as we and others have previously reported [23,34,42]. Additionally, we also observed that promoter-based hypomethylation was always associated with increased gene expression, suggesting that as previously reported, body based and promoter based methylation have differing functional consequences on gene expression [11,34,42]. These data suggest that measures of gene expression are critical to determine whether CpG methylation has a functional consequence and whether it represents a silencing or activating mechanism, highlighting the complex relationship between CpG methylation and gene expression.

While we have found CpG methylation control of key biological pathways, this study is not without limitations. A relatively small sample size (n = 36) was available for study and BMI, a risk factor for preeclampsia, was not available and therefore could not be accounted for. Lastly, preeclamptic women and normotensive women differed significantly in gestational age. To address this limitation, we included gestational age as a covariate in the analytical model as well as compared the identified DMGs/fDMGs to a known database of genes changed in expression associated with gestational age resulting in little overlap. Nevertheless, this study has numerous advantages such as the utilization of placental tissue versus maternal serum, which is key as poor placentation is highly relevant in relationship to placental toxicity.

Taken together, out study provides evidence that altered DNA methylation of the TGF-β pathway is linked to its elevated expression in the preeclamptic placenta. These data increase the understanding of mechanisms of transcriptional control that underlie preeclampsia and provide new target molecules and biological pathways in the formulation of preventative and treatment strategies.

## Supporting Information

S1 Table Probes (n = 389) located in (or 10 base pairs away from) SNPs associated with preeclampsia. (XLSX)

S2 Table Table of differentially methylated probes (DMPs) (n = 989). (XLSX)

S3 Table Preeclampsia-associated differentially methylated CpG sites with significant association to gene expression levels (n = 132). (XLSX)

S4 Table Preeclampsia-associated genes (n = 376) identified through literature search. (XLSX)

S5 Table Canonical pathways identified in association with differentially methylated genes (DMGs), functionally changed DMGs (fDMGs), negatively correlated fDMGs, and positively correlated fDMGs. (XLSX)

S6 Table Differentially expressed genes (DEGs) (n = 729), independent of methylation status, associated with preeclampsia. (XLSX)
